# The Feasibility of Using Yellow Mealworms (*Tenebrio molitor*): Towards a Sustainable Aquafeed Industry

**DOI:** 10.3390/ani11030811

**Published:** 2021-03-13

**Authors:** Laiba Shafique, Hany M. R. Abdel-Latif, Faiz-ul Hassan, Mahmoud Alagawany, Mohammed A. E. Naiel, Mahmoud A. O. Dawood, Sevdan Yilmaz, Qingyou Liu

**Affiliations:** 1 State Key Laboratory for Conservation and Utilization of Subtropical Agro-Bioresources, Guangxi University, Nanning 530005, China; laibazoologist@gmail.com; 2 Department of Poultry and Fish Diseases, Faculty of Veterinary Medicine, Alexandria University, Alexandria 22758, Egypt; hmhany@alexu.edu.eg; 3 Institute of Animal and Dairy Sciences, University of Agriculture, Faisalabad 38040, Pakistan; f.hassan@uaf.edu.pk; 4 Poultry Department, Faculty of Agriculture, Zagazig University, Zagazig 44519, Egypt; dr.mahmoud.alagwany@gmail.com; 5 Department of Animal Production, Faculty of Agriculture, Zagazig University, Zagazig 44511, Egypt; mohammednaiel1984@gmail.com; 6 Department of Animal Production, Faculty of Agriculture, Kafrelsheikh University, Kafrelsheikh 33516, Egypt; mahmouddawood55@gmail.com; 7 Department of Aquaculture, Faculty of Marine Sciences and Technology, Canakkale Onsekiz Mart University, 17100 Canakkale, Turkey; sevdanyilmaz@comu.edu.tr

**Keywords:** fish meal, aquafeed, insects, growth, body composition, fish health

## Abstract

**Simple Summary:**

The expansion of the aquaculture industry depends mainly on aquafeed availability at reasonable prices. The common ingredients of aquafeed (e.g., fish and soybean meals) are not sustainable due to a lack of resources and increasing prices. Seeking alternative non-traditional ingredients is among the choices of nutritionists to produce high-quality feed at a feasible cost. Yellow mealworms (*Tenebrio molitor*) (TM) have been introduced to the feed industry as protein sources of a circular economy. Many studies have investigated the possibility of including *T. molitor* meals as a substitute for fish and soybean meals in aquafeed. Thus, this review exclusively presents an assemblage of the literature on the possibility of including *T. molitor* in aquafeed as a suggestion for the sustainability of the aquaculture industry.

**Abstract:**

The success of the aquafeed industry mainly depends on the availability of raw ingredients with high nutritional value, such as fishmeal (FM). However, the increased demand for FM elevates its prices and leads to high feed costs. Thus, there is an urgent need to find suitable alternatives for FM in fish diets to achieve sustainability in aquaculture. Currently, attention is being paid to the possibility of using insect meals as FM substitutes in aquafeed because of their relatively high nutritional quality. TM is one of those insects that can be regarded as a unique candidate because of its relatively high nutritional value. TM are rich sources of essential amino acids (methionine), lipids, and fatty acids, which vary based on the developmental stage of the worms. Although TM have an abundant amount of chitin as a fiber source and other anti-nutritional factors, numerous studies have investigated the efficacy of partial or complete substitution of FM by *T. molitor* in fish diets. In this context, we reviewed the current research findings on the achievable inclusion levels of *T. molitor* versus FM substitution in the diets of several finfish and shellfish species. We discussed the potential use of *T. molitor* as an FM substitute in fish diets and evaluated its effects on growth, biometric indices, and body composition. Besides, the hematological parameters, immunological responses, antioxidative efficacy, intestinal health status, and sensory criteria of fish fed *T. molitor*-based diets were also assessed.

## 1. Introduction

According to the International Feed Industry Federation (IFIF), the world population will be more than 10 billion by 2050. For this purpose, a double amount of animal protein is required for nutrition and food security. The Food and Agriculture Organization (FAO) emphasizes that finding alternative ways of feeding aquatic animals is imperative since fulfilling the increasing demands based on fish meal (FM) and fish oil (FO) resources might be impossible [1].

FM is one of the most critical components of aquafeed because of its exceptional nutritional value of highly digestible proteins and essential amino acids (EAAs) and its palatability when included in fish diets [2,3,4]. The increased demand for FM and the decrease in its availability will, in turn, increase its price for farmers. Thus, there is an urgent need to find new alternatives for partial or total FM replacement in fish diets, which should be less expensive, environmentally friendly, relatively safe, sustainable, and palatable for fish species [5,6]. Among these alternatives, insect meals have attracted the vision of researchers as a relatively available and cheap protein with high nutritional value [7,8,9,10]. 

There are plentiful benefits of using insect meals in fish diets, including (a) the abundance of EAAs and fatty acid contents [11], (b) less competition for land resources compared with other feed ingredients [12], (c) fast growth and the ability for use of biowastes with increased feed conversion efficiency [13], and (d) simple reproduction [14]. Thus, some important factors should be taken into consideration for proper selection of the type of insect meals before including them into aquafeeds, such as the suitability of lipids and the fatty acid content [15], the EAA profile [7], digestibility [16], and low contamination of mycotoxins [17]. More importantly, several other factors should be considered, too, limiting the usage of insect meals, such as relatively higher prices, their safety margin during dietary inclusion, and, finally, the human consumer acceptability.

The yellow mealworm (*Tenebrio molitor* (TM)) (family Tenebrionidae) is one of the unique candidates as a potential protein source for possible FM replacement in aquafeeds [18]. The larvae and pupae of *T. molitor* have a considerable content of proteins and lipids [19] as they contain several essential amino acids (EAAs) (especially methionine), lipids, and essential fatty acids (EFAs), which vary based on the developmental stage of the worms. It is well known that although TM have a considerable protein and lipid content, TM, as well as other insect species, have deficiencies in some amino acids and fatty acids.

The use of *T. molitor* for partial or complete substitution of FM in aquafeed has been extensively assessed, on a scientific basis, according to its influence on fish growth and nutrient use in a wide range of finfish species such as yellow catfish (*Pelteobagrus fulvidraco*) [20], Nile tilapia (*Oreochromis niloticus*) [17], rainbow trout (*Oncorhynchus mykiss*) [21,22], European sea bass (*Dicentrarchus labrax*) [23,24,25], gilthead sea bream (*Sparus aurata*) [26,27], Atlantic salmon (*Salmo salar*) [28], and red seabream (*Pargus major*) [29] among others, and also in shellfish species such as the giant freshwater prawn (*Macrobrachium rosenbergii*) [30,31].

The overarching goals of this work are to provide a comprehensive review to summarize the current knowledge and research findings on the effects of dietary substitution of *T. molitor* on the overall performance of several finfish and shellfish species and mainly focus on the following points: (a) the possibility of TM for partial or complete replacement of FM in aquafeed; (b) evaluation of the nutritional effects of dietary TM on growth, feed use, body composition, and gut health; (c) discussion of the potential impacts of dietary TM on the overall health status in terms of hematobiochemical indices, antioxidant potential, and immunological responses; and (d) sensory criteria of fillets of fish fed TM-based diets.

## 2. Nutritive Value of the Yellow Mealworm: A Brief Overview

The nutritional composition of *T. molitor* varies according to their developmental stage [14,32]. According to some previously published data, the proximate chemical composition evaluation of *T. molitor* larvae is illustrated in Table 1. Moreover, Table 2 and Table 3 provide a summary of the amino acid (AA) (essential and non-essential AAs) and fatty acid (FA) (saturated, monounsaturated, and polyunsaturated FAs) profiles of *T. molitor*. 

In brief, TA are rich in crude protein (about 47–60% of dry matter (DM)) and crude lipids (about 31–43% of the DM), along with a relatively low ash percentage (<5% of the DM) and calcium (Ca) content [33]. Regarding their AA profile, TM have some AA deficiencies, such as methionine, threonine, lysine, histidine, and cysteine, while they are rich in valine and tyrosine [11]. More importantly, the developmental stages of *T. molitor* larvae might affect their AA composition [34].

## 3. Dietary Inclusion *T. molitor* with Possibility of FM Replacement 

The efficacy of *T. molitor* for partial or complete dietary substitution of fishmeal in practical fish diets has been previously reviewed and described [7,13]. This review article evaluates the effects of the inclusion of TM in aquafeed on the overall health status and performance of the treated fish (Table 4, Table 5, Table 6, Table 7 and Table 8).

It has been found that the inclusion levels of TM in aquafeed depends on several factors, for instance, the feeding habits, fish size, and growing stage [21]. In this regard, carnivorous species probably cannot accept high TM levels, while omnivorous species can accept high levels of TM as an FM replacer [13]. Another limiting factor is the processing of TM (either full fat or mechanically defatted), which could also affect the acceptability of fish for partial or total replacement of FM with TM meals. It has been reported that the high lipid content of TM reduces the availability of crude protein and lowers the extrusion quality during feed processing [48]. Thus, defatted TM could be highly recommended to avoid the degradation and unstable pelleting process of aquafeed. The following sections offer the main outputs of studies that investigated the possibility of including TM in relation to the dietary requirements for different fish species.

### 3.1. Rainbow Trout (Oncorhynchus mykiss)

TM larvae meal could be included up to 50% in rainbow trout diets at the expense of FM [45,49,50]. However, defatted TM larvae could substitute up to 100% of dietary FM [48,51]. In contrast, Iaconisi, et al. [52], Iaconisi, et al. [53], and Valipour, et al. [54] indicated that full-fat TM larvae could be included not more than 25% in trout diets. However, Henry, et al. [55] demonstrated that 50% dietary inclusion of full-fat TM larvae could replace up to 67% of FM in trout feeds without negative impacts on trout growth. Harsij, et al. [56] reported that live TM worms could be included in up to 60% of diets, and Antonopoulou, et al. [57] demonstrated that full-fat TM larvae could be included up to 60% in diets. Recent studies have suggested that 20% of trout diets can be successfully substituted by full-fat TM larvae [58] and partially defatted TM larvae [21] without undesirable effects on the treated fish. Jeong, et al. [22] also reported that 14% of full-fat TM larvae could be included in trout diets.

### 3.2. European Seabass (Dicentrarchus labrax)

Gasco, et al. [59] suggested the possibility of dietary inclusion of 25% of TM in diets of *D. labrax*, with no harmful influence on the growth rate. A study subsequently conducted by Antonopoulou, et al. [57] showed that 50% of FM replaced with full-fat TM larvae resulted in considerable improvement of the gut bacterial diversity of the treated *D. labrax*. The feeding trial of Basto, et al. [60] indicated the potential ability of defatted TM larvae to substitute up to 80% of FM in *D. labrax* diets. A recent study by Mastoraki, et al. [23] suggested that 30% of FM can be effectively substituted with whole TM larvae, with no harmful impacts on the fish growth performance. Interestingly, Reyes, et al. [61] reported that substitution of less than 50% of FM in sea bass diets with TM larvae did not influence on growth indices or proximate analysis of the fillets.

### 3.3. Sparidae Species

Piccolo, et al. [27] reported that 25% of FM could be successfully substituted with TM larvae, without any harmful impacts on the growth or whole-body composition of gilthead seabream (*Sparus aurata*). Moreover, dietary inclusion of 25% full-fat TM larvae could replace 35% of FM in gilthead seabream diets [26]. On the other hand, Antonopoulou, et al. [57] reported a substantial improvement of the gut bacterial diversity when 50% of FM in fish diets was substituted with full-fat TM larvae. In a similar sense, Iaconisi, et al. [53] also illustrated that 50% dietary inclusion of full-fat TM larvae positively impacted the AA composition of the fish body.

Iaconisi, et al. [18] showed that 25% of FM could be successfully substituted with full-fat TM larvae in blackspot seabream (*Pagellus bogaraveo*) diets, with no side effects on the growth or proximate composition of fish fillets. On the other hand, it was noticed that up to 100% of FM in the diets of the red sea bream (*Pargus major*) could be successfully substituted with defatted TM larvae, with a positive impact on fish growth performance [29].

### 3.4. Clariidae Species

TM larvae meal powder could substitute up to 60% of FM, with no effects on the growth parameters or feed use indices of African catfish (*Clarias gariepinus*) [62]. However, higher inclusion doses of TM (about 35–43%, which is equal to 80–100% FM substitution) induced a reduction in fish growth, feed use, and protein efficiency ratios. More importantly, Roncarati, et al. [63] found that total substitution of dietary FM with dried TM larvae substantially decreased the growth of common catfish (*Ameiurus melas*) compared to the control group that was fed a diet with 50% FM. The study conducted by Su, et al. [20] reported that up to 75% of FM could be efficaciously substituted with TM in the diets of yellow catfish (*Pelteobagrus fulvidraco*), with no differences in the growth indices and whole-body composition compared to the FM group.

### 3.5. Other Fish Species

It was found that up to 50% of FM can be substituted in Nile tilapia (*Oreochromis niloticus*) diets with TM larvae, with no differences in the whole-body composition [17]. Kim, et al. [64] showed that 17% of FM could be substituted with TM larvae in diets of the Japanese flounder (*Paralichthys olivaceus*), with better growth performance. Another study that was reported by Song, et al. [65] exemplified that 12.3% of FM (corresponding to 4.92% of TM inclusion in diets) could be efficiently replaced with TM worms in the diets of the Pearl gentian grouper (*Epinephelus lanceolatus* ♂ × *E. fuscoguttatus* ♀), with no side effects on growth and survival rates. Recently, Józefiak, et al. [66] reported no significant impact in the growth indices of the Siberian sturgeon (*Acipenser baerii*) when 15% of dietary FM was replaced with TM larvae. Redman, et al. [67] reported that 25% of FM could be successfully substituted in the diets of the black sea bass (*Centropristis striata*) with TM, with positive impacts on the fish growth performance.

### 3.6. Shrimp

Replacement of 50% and 100% of dietary FM by TM larvae would be suitable in the diets of the Pacific white shrimp (*Litopenaeus vannamei*), without negative impacts on shrimp growth rates [68]. Similarly, it was suggested that 100% of FM in white shrimp diets could be successfully substituted by dehydrated TM larvae [69]. Panini, et al. [70] also reported the significant capability of TM to replace 100% of FM in the diets of white shrimp. Likewise, dried super-TM have also been effectively used to replace 50% of FM in white shrimp diets, without adverse effects on growth [31]. Motte, et al. [71] also found that defatted TM larvae could potentially substitute 50% of FM in white shrimp diets, without impacts on the survival rate among all groups compared with controls.

From the aforementioned studies, we concluded that the TM levels required for partial or complete replacement of FM in fish diets are closely related to the processing of TM larvae prior to dietary inclusion. It was noticed that defatted TM larvae could replace the whole FM content in diets, and these finding highlights the importance of the processing of TM meals before inclusion in fish diets. Moreover, the recommended dietary inclusion level of TM to substitute FM varies between the same fish species according to fish size and growth stage to fulfill the protein requirements in each life stage [7]. Other limiting factors were also reported by Roncarati, et al. [63], who found that the chitin content, digestibility, AA balance, and FA composition are the main factors that affect the inclusion levels of TM in fish diets. Besides, Khosravi, et al. [72] and Sankian, et al. [73] also suggested that these discrepancies might be attributed to diet formulation and culture conditions.

## 4. Effects of Dietary Supplementation with *T. molitor*

Table 4, Table 5, Table 6, Table 7 and Table 8 summarize the effects of dietary inclusion of *T. molitor* on the overall performance of a wide variety of finfish and shellfish species.

### 4.1. Growth Performance

#### 4.1.1. European Seabass

It was found that dietary inclusion of 25% TM larvae in the diets of European seabass did not show any adverse effect on weight gain (WG); meanwhile, dietary inclusion of 50% TM larvae resulted in growth reduction and less favorable outcomes on the specific growth rate (SGR), feed conversion ratio (FCR), protein efficiency ratio (PER), and feed intake (FI) of treated fish [59]. Similarly, Gasco, et al. [38] found that inclusion of 50% full-fat TM larvae in the diets of European seabass juveniles for 70 days induced negative impacts on the final body weight (FBW), WG, SGR, and FI compared to fish fed FM-based diets. However, Reyes, et al. [61] recorded no significant differences in the FCR and PER in *D. labrax* fed for 49 days a diet with 50% of FM replaced with TM larvae compared with the FM-based group. Mastoraki, et al. [23] reported no significant differences in the SGR, FI, viscerosomatic index (VSI), hepatosomatic index (HSI), and relative gut length (RGL), alongside a slight increase in the FCR, in *D. labrax* fed diets with 30% of FM replaced with whole TM larvae for 84 days compared with the FM group.

#### 4.1.2. Sparids

Piccolo, et al. [27] reported no negative effects on the WG and FBW of gilthead seabream fed diets with 25% of FM replaced with TM larvae. alongside a slight decrease in the PER and FCR compared with the FM-supplemented group. However, these authors indicated a reduction in fish growth, with less favorable impacts on the SGR, FCR, and PER in fish groups fed diets with 50% of FM replaced with TM larvae. Piccolo, et al. [26] reported a higher FBW, SGR, WG%, and PER, alongside a lower FCR, in gilthead seabream fed diets in which 25% of FM was replaced with full-fat TM larvae. 

Iaconisi, et al. [18] reported no considerable variations in the FI, FCR, and SGR in blackspot sea bream groups fed TM-based diets with 25% and 50% of dietary FM replaced compared with those fed FM-based diets. Another different finding was indicated by Ido, et al. [29], who found that the highest FBW, SGR, and WG were observed in red seabream fed diets with 100% of FM substituted with defatted TM larvae for 28 days, alongside no significant differences observed in the FCR and FI among all fish groups.

#### 4.1.3. Salmonids

Saravanan, et al. [51] found that the FBW of rainbow trout fed diets with total replacement (100%) of FM with mechanically defatted TM larvae for 60 days was showed an eightfold increase compared to their initial body weight (IBW). These authors also noticed that dietary TM inclusion resulted in a decrease in the FCR and FI, alongside a rise in the PER, with increasing dietary inclusion levels of TM larvae. Another study by Belforti, et al. [45] demonstrated that the FBW and WG of rainbow trout were not significantly affected among rainbow trout fed diets supplemented with 25% and 50% of full-fat TM larvae for 90 days.

Valipour, et al. [54] reported the highest FBW in rainbow trout fed diets with 25% of FM replaced with TM larvae powder. However, a better FCR was noticed in the fish group fed a diet with 75% FM replacement with TM larvae. Moreover, Józefiak, et al. [58] found a significant increase in the WG in rainbow trout fed 20% full-fat TM larvae-based diets. However, no differences were detected in the FCR, SGR, and PER in TM groups versus the controls. Contrarily, Rema, et al. [48] reported a substantial increase in the FBW, FI, SGR, and PER and a better FCR in rainbow trout fed diets with 100% of FM substituted with defatted TM larvae. On the other hand, Chemello, et al. [21] described no significant differences in the SGR, FCR, PER, FI, FBW, VSI, and WG among rainbow trout fed 20% TM-based diets (with 100% FM replacement) compared with the FM group. However, the authors recorded the highest HSI values in the fish group fed diets with total replacement of FM with partially defatted TM larvae.

Jeong, et al. [22] suggested that the WG and SGR increased in rainbow trout fed diets supplemented with 14% full-fat TM larvae and then decreased with increasing FM substitution levels over 14%. These authors further reported that the highest PER and the lowest FCR were found in all TM-supplemented groups, with no considerable divergences in the condition factor (CF), VSI, and HSI between groups. Hoffmann, et al. [42] revealed the highest HSI and VSI in sea trout (*Salmo trutta m. trutta*) fed diets supplemented with 20% enzymatically hydrolyzed and non-processed full-fat TM larvae compared with the FM group. However, the highest FBW and WG were noticed in the FM group.

#### 4.1.4. Other Fish Species

Ng, et al. [62] recorded the highest FBW, WG%, SGR, PER, and net protein utilization percentage (NPU%) in African catfish fed diets with 20% of FM substituted with TM powder. Meanwhile, a slight depression in the FBW, SGR, FER, PER, and NPU% was noticed in catfish groups fed solely on live TM. Conversely, Roncarati, et al. [63] demonstrated a significant decrease in the FBW in common catfish fed a diet with 50% of FM replaced with TM compared to the FM group, with no significant changes in the FCR among all experimental groups. However, Su, et al. [20] reported no noticeable differences in the FI, SGR, and feed conversion efficiency (FCE) among yellow catfish fed TM-based diets with up to 75% FM replacement compared with those fed FM-based diets.

Kim, et al. [64] reported that Japanese flounder fed diets with 17% of FM substituted with TM larvae had a significantly higher WG and SGR, a better FCR, and no significant effect on the HSI. Interestingly, Khosravi, et al. [72] reported a significant increase in the WG, protein retention (PR), and SGR of rockfish fed diets supplemented with not more than 16% TM. Moreover, a significant decrease in the WG, PR, and SGR was noticed by increasing the dietary inclusion levels of TM by over 16% to 32%.

Redman, et al. [67] reported a significant elevation in the FBW, WG, FI, and SGR in black sea bass fed diets with 25% of FM substituted with TM. Similarly, Sankian, et al. [73] reported a significant elevation in the WG, SGR, FER, PER, and PR in mandarin fish fed diets supplemented with 20% full-fat TM larvae at the expense of FM and then decreased afterward with an increase in the inclusion dose over 20%. Moreover, no considerable changes were observed in the FI, survival rate percentage (SR%), HSI, VSI, and CF among all experimental groups. On the other hand, Józefiak, et al. [66] demonstrated no differences in the FBW, WG, FCR, SGR, PER, and SR% in Siberian sturgeon fed diets with 15% of FM substituted with TM larvae.

Sánchez-Muros, et al. [17] recorded a significant decrease in the FBW, FCE, PER, and daily growth coefficient (DGC) in Nile tilapia fed TM-based diets with 50% FM replacement, alongside no significant alterations in the FI and CF among groups. On the contrary, Tubin, et al. [74] reported a linear increase in the FWB, WG, SGR, FCR, and HSI in Nile tilapia raised in the biofloc system, along with an increase in the dietary inclusion levels of TM by up to 10%. Recently, Xu, et al. [75] reported a significant increase in the FCR and FI, alongside no alterations in the general relative intestine length (GRL), HSI, and VSI in mirror carps (*Cyprinus carpio* var. specularis) fed diets supplemented with 25 g of yellow mealworm oil (YMWO)/kg diet for 59 days compared with the group fed diets supplemented with 25 g of black solider fly larvae oil (BSFLO)/kg diet.

#### 4.1.5. Shrimp

Chung et al. [68] indicated that Pacific white shrimp fed diets with 50% and 100% of FM replaced with TM larvae had significantly higher WG and SGR values with a better FCR than the FM group. However, Panini, et al. [69] observed that the WG, SGR, FI, and FCR were not affected by dietary TM larvae inclusion up to 100% of dietary FM in shrimp diets.

Recently, Motte, et al. [71] reported a significant increase in the FBW, WG, and average daily gain (ADG) with the best FCR in white shrimp fed diets with 50% of FM replaced with defatted TM larvae. The authors also noticed that the FI and PER were not considerably affected among TM and FM groups. Feng, et al. [30] elucidated that inclusion of 12% TM protein in the diets of giant freshwater shrimp (*Macrobrachium rosenbergii*) resulted in a significant improvement of the WG, WG%, SGR, and PER compared with the FM group; meanwhile, no significant changes were recorded in the FCR and survival rate between all groups.

From the previously mentioned studies, it can be suggested that discrepancies in the growth performance of fish species fed diets with FM substituted with graded levels of TM might be accredited to several factors, including:

(a) TM factors such as quality, processing, chitin content, proximate chemical composition analysis (CF, crude lipids (CP), crude protein (CL), and gross energy (GE)), and their FA and AA profiles;
(b) fish factors such as size, age, growth stage, and ability to digest insect chitin (presence of chitinase enzyme);
(c) dietary factors (level of FM substituted with TM and proximate composition of diets); and
(d) experimental conditions such as water temperature, rearing system, and the experimental setup, etc.

Edible insect meal such as TM is widely known for its high-quality protein and unique nutritional characteristics [76]. The nutritional components of TM include a relatively increased amount of CP (53.2%) and CL (34.5%) [20], which can enhance the growth parameters of fish [76]. Besides, the improvement of the nutrient use efficiency by fish species might also be due to chitinous materials in fish diets. These chitinous materials can modulate the gastro-intestinal tract (GIT) microbiota, leading to improved fish growth, when being included in relatively adequate amounts [57].

The depression of growth performance parameters and feed and protein use indices may be linked with the higher inclusion levels of TM because of chitin found in the exoskeletons of the mealworms [62]. For a better discussion of this point, there are species-specific differences in the presence of the chitinase enzyme, which can help to digest chitin. For instance, marine fish can easily digest chitinase because of their chitinase activities in their digestive tracts [77]. On the other hand, the presence of the chitinase enzyme is relatively reduced or completely absent in some freshwater fish, so they cannot digest chitin [13]. In this regard, it was found that Nile tilapia (*O. niloticus* × *O. aureus*) fed a diet with 2% chitin displayed lower growth rates and FER [78]. Moreover, chitin is inadequately digested by numerous fish species leading to a slower growth rate [79,80,81].

### 4.2. Proximate Body Composition, FAs, and AA Profile of Fillets

This section evaluates the impacts of dietary inclusion of TM on the whole-body proximate constituents, FAs, and AA profiles in fillets of several fish species.

#### 4.2.1. European Seabass

Gasco, et al. [59] found no significant differences in the whole-body proximate analysis of *D. labrax* fed diets supplemented with 25% and 50% TM larvae. These authors also reported decreased docosahexaenoic acid (DHA) and eicosapentaenoic acid (EPA) fatty acids in fish fed diets supplemented with 50% TM larvae. Basto, et al. [60] reported an increase in the linoleic acid content in muscles, with no impacts on the contents of EPA and DHA in the muscles of *D. labrax* fed diets supplemented with defatted TM larvae at the expense of 80% of dietary FM. Gasco, et al. [38] also recorded no significant effects of 25% and 50% TM-based diets on the CP content and ether extract (EE) of *D. labrax*. These authors further reported an increase in the C18:2 n6 content, alongside a decrease in the C20:5n3 and C22:6 n3 content, in 25% and 50% TM groups. Reyes, et al. [61] found no significant variations in the proximate composition of *D. labrax* fillets fed diets with 50% of FM replaced with TM larvae. Conversely, Mastoraki, et al. [23] reported a significant decrease in the CP, DM, EE, and GE, alongside the highest n-6 polyunsaturated fatty acid (PUFA) content, in *D. labrax* fed diets with 30% of FM replaced with whole TM larvae.

#### 4.2.2. Sparids

Piccolo, et al. [27] demonstrated no change in the whole-body composition in gilthead seabream fed diets with 25% and 50% of the FM replaced with TM larvae. Iaconisi, et al. [18] reported the same findings in blackspot seabream fed diets with 25% and 50% of FM was replaced with full-fat TM larvae. These authors also found a significant increase in linoleic acid with increasing dietary full-fat TM larvae levels. Moreover, Iaconisi, et al. [53] found higher alanine, leucine, lysine, arginine, leucine, glycine, and proline levels in gilthead seabream fed diets with 50% of FM replaced with full-fat TM larvae. The authors also demonstrated lower histidine, taurine, and phenylalanine levels in groups fed diets with 25% and 50% of FM replaced with TM larvae. Therefore, the AA profile of the muscles of gilthead sea bream fed diets with FM substituted with TM depends on the percentage of substitution and might require fine-tuning of the feed formulation, particularly AAs, to avoid deficiencies.

#### 4.2.3. Salmonids

There were no significant differences in the whole-body composition of rainbow trout fed TM-based diets than those fed FM-based diets [22,48]. Conversely, Belforti, et al. [45] demonstrated a significant increase in the CP content and decreased CL in rainbow trout fillets of fish fed diets with 25% and 50% TM over those in the FM group. Moreover, Harsij, et al. [56] found the highest CP and CL in fillets of rainbow trout fed diets supplemented with up to 60% TM. Rema, et al. [48] reported a noticeable increase in the retention of protein, phosphorus, and energy in rainbow trout fed TM-based diets with up to 100% FM replacement.

Iaconisi, et al. [52] reported an elevation in C16:0, C18:1n9, and C18:2n6 levels, alongside a decrease in the EPA and DHA content, in trout fillets with increasing TM inclusion levels by up to 50% of diets. Moreover, these authors found a gradual decline in the PUFA/saturated fatty acids (SFA) and n3/n6 ratio with increasing dietary inclusion levels of TM larvae. Moreover, Iaconisi, et al. [53] illustrated significantly increased alanine, taurine, tyrosine, cysteine, leucine, and proline levels in rainbow trout fed diets with 50% full-fat TM larvae. However, there was a significant decrease in glutamine, glycine, histidine, histidine, methionine, and threonine levels in the same group. Therefore, the AA profile in the muscles of salmonids fed diets with FM substituted with TM depends on the percentage of substitution and might require fine-tuning of the feed formulation, particularly AAs, to avoid deficiencies.

#### 4.2.4. Other Fish Species

Khosravi, et al. [72] demonstrated no impacts of dietary supplementation with up to 32% of TM on the whole-body composition, fillet analysis, and EAA composition of rockfish (*Sebastes schlegeli*). In a similar sense, Sankian, et al. [73] reported no differences in the whole-body analysis of mandarin fish fed diets with up to 30% of full-fat TM-larvae. However, higher monosaturated fatty acids (MUFA) and lower PUFA were recorded in TM-supplemented groups than the control group. Sánchez-Muros, et al. [17] also indicated no significant differences in the whole-body composition of Nile tilapia fed TM-based diets to replace up to 50% of FM compared to the control group. However, Tubin, et al. [74] reported a linear increase in the DM and EE in the carcass composition of Nile tilapia raised in a biofloc system with increased dietary inclusion levels of TM by up to 20% of fish diets. Recently, Xu, et al. [75] reported no variations in the whole-body composition of mirror carps fed diets supplemented with 25% yellow mealworm oil compared to BSFLO-supplemented diets. On the contrary, Ng, et al. [62] reported the highest CP content in the bodies of African catfish fed diets with FM replaced with 20% of TM powder. However, a higher whole-body CL content was recorded in catfish fed solely live TM.

#### 4.2.5. Shrimp

Panini, et al. [70] and Panini, et al. [69] demonstrated no significant differences in ash and CP levels in the muscles of shrimp fed TM-based diets with up to 100% of FM substitution and recorded a linear elevation in the shrimp body lipid content with increasing dietary TM levels. Interestingly, Panini, et al. [69] indicated a linear decrease in the EPA and DHA content in shrimp muscles with increasing TM in diets. On the other hand, Feng, et al. [30] reported opposite findings in *M. rosenbergii* fed diets supplemented with graded TM protein levels, whereas there was a linear increase in the CL with a decrease in the CP in the carcass and muscle with increasing dietary inclusion of TM protein.

From the previously reported literature, discrepancies in the proximate composition analysis of the fish body could be accredited to numerous factors, including a) fish age and size, b) composition and nutritional values of the diet, c) source and quality of TM, d) form of TM larvae used (full fat, partially defatted, or defatted), and e) experimental conditions. The FA and AA profiles of fish generally reflect the dietary FA and AA content of TM. These findings were in line with those demonstrated by Gómez-Requeni, et al. [82], who reported an elevation in the AAs in the muscles of gilthead sea bream fed diets with FM replaced with increasing levels of plant protein sources. 

## 5. Apparent Digestibility of TM-Based Diets

Gasco, et al. [38] illustrated that the apparent digestibility coefficient (ADC) of the CP in seabass diets containing 25% TM notably increased than that in the FM group. Basto, et al. [24] concluded that the ADCs of the DM, CP, CL, GE, and phosphorus of TM-based diets have the highest values, and these diets contain a highly digestible total EAA content (>89%).

Piccolo, et al. [26] reported that the coefficients of the total tract apparent digestibility of the EE and CP of diets were lower in gilthead seabream fed diets with 50% of FM replaced with full-fat TM larvae compared with the control group. Moreover, Panini, et al. [70] illuminated that the ADC of the DM, GE, CP, and EAAs of shrimp diets supplemented with 15% dehydrated TM larvae were 45.9%, 66.5%, 76.1%, and 72–86%, respectively.

Belforti, et al. [45] recorded a significant decline in the ADC of the CP in the diets of rainbow trout fed diets supplemented with 50% full-fat TM larvae compared with the control group; however, the ADC of the DM and CL were not affected. On the other hand, there was a significant increase in the ADC of the CP in the diets of rainbow trout fed diets with 25% of FM replaced with partially defatted TM larvae compared with 50% and 100% FM replacement groups [21]. Conversely, Rema, et al. [48] demonstrated no significant variations in the ADC of the DM, CP, CL, phosphorus, and GE in rainbow trout fed diets with graded levels of TM larvae. These discrepancies in the ADC of dietary ingredients may be attributed to the proximate composition analysis of the TM included in fish diets.

Finke [83] illustrated that chitin in the crude fiber, nitrogen-free extract, and possibly AAs from proteins bound to chitin can decrease the ADC of the insect DM. A similar study conducted by Yang, et al. [84] found that the digestibility of the DM in dietary ingredients consumed by shrimp tended to decline with the increase in the chitinous content of the ingredients. Moreover, it was demonstrated that the low ADC of the GE in TM possibly increased because of the CL content in TM and their FA composition [70].

Marono, et al. [85] proposed that chitin in TM may participate in reducing the ADC of the CP through decreasing the gut transit time and physically shielding the protein from enzymatic degradation. Chitinase activity has been demonstrated in the digestive tract of several marine fish species [86], thus proposing that these marine fish can degrade chitin [77]. Differently, chitinase enzyme activity is relatively reduced or completely deficient in rainbow trout [13]. This could clarify the decline in the ADC of the CP in this fish species when fed diets supplemented with the highest inclusion levels of TM. Thus, dietary supplementation with exogenous enzymes is required.

## 6. Hematobiochemical Parameters

Generally, fish hematological and serum biochemical indices are considered vital measurements and biological indicators for evaluating the general health status, hepatorenal functions [87,88], and physiological stress responses of fish fed formulated diets [89,90].

Jeong, et al. [91] and Khosravi, et al. [72] reported no significant variations in plasma total protein (TP), aspartate aminotransferase (AST), alanine aminotransferase (ALT), triglycerides (TG), total bilirubin (TBIL), albumin (ALB), and total cholesterol (TCHO) in rockfish fed diets supplemented with up to 32% TM larvae. A study conducted by Sankian, et al. [73] also showed no significant differences in the TP, ALB, ALT, AST, TG, and alkaline phosphatase (ALP) in Mandarin fish fed diets supplemented with up to 30% full-fat TM larvae at the expense of FM. However, these authors recorded a significant decrease in plasma TCHO in the group fed diets with 30% of FM replaced with TM larvae.

Valipour, et al. [54] demonstrated no considerable differences in erythrocyte parameters such as the red blood cell (RBC) count, mean corpuscular hemoglobin concentration (MCHC), mean corpuscular volume (MCV), and plasma ALT, AST, TG, glucose (GLU), low-density lipoprotein (LDL), and high-density lipoprotein (HDL) values among rainbow trout groups fed diets with TM powder to replace up to 100% of dietary FM. Meanwhile, these authors demonstrated a significant reduction in hemoglobin (Hb%), hematocrit (HTC), and cholesterol (CHO) levels with increasing TM levels to replace FM in the diets of rainbow trout.

Xu, et al. [75] recorded no differences in ALB, TP, ALT, AST, ALP, TCHO, and TG levels in mirror carps fed diets supplemented with YMWO (25 g/kg diet) compared with BSFLO and FM groups. The same results were shown by Hoffmann, et al. [42], who found no significant differences in hepatic ALT and AST in sea trout groups fed diets supplemented with 20% enzymatically hydrolyzed non-processed full-fat TM larvae.

Jeong, et al. [22] elucidated no significant differences in ALT, AST, ALP, ALB, TP, TCHO, and TBIL levels among rainbow trout fed different levels of full-fat TM larvae replacing FM in their diets compared to the FM group. Mastoraki, et al. [23] also reported no significant differences in plasma phospholipids, AST, ALT, and glutamate dehydrogenase (GDH) activities of *D. labrax* fed diets supplemented with several levels of TM compared to controls. Meanwhile, *D. labrax* fed with TM diets exhibited a significant decrease in plasma GLU, CHO, lactate, and TG values compared to the FM group.

According to previous studies, no changes are found in most hematological and serum biochemical indices studied in fish species fed TM-based diets. These findings confirm that the dietary inclusion of TM does not negatively affect the general health condition of fish. Some studies reported a significant reduction in serum CHO and TCHO levels in fish fed diets supplemented with TM [23,54,73]. A similar finding was reported in Jian carp (*Cyprinus carpio* var. Jian) fed a diet supplemented with 68–90% of silkworm pupae [92]. Besides, Magalhães, et al. [93] demonstrated a marked reduction in plasma TCHO levels in European sea bass fed diets with high levels of dietary *Hermetia illucens* pre-pupae meal. The reason for that was previously defined by Khoushab and Yamabhai [94], who indicated that chitin and chitooligosaccharides (chitosan) could decrease the hepatic and plasma TG and TCHO levels through interruption of the enterohepatic circulation of bile, hindering the normal digestion and absorption of lipids through the intestinal tract and hampering FA biosynthesis in the hepatocytes, resulting in hypolipidemic and hypocholesterolemic conditions in fish and animals.

## 7. Antioxidative Capacity

Oxidative stress arises because of the over-production of reactive oxygen species (ROS) such as hydroxyl radicals and superoxide anion radicals, which subsequently induces risky and damaging impacts on the fish body, such as DNA damage; disruption of the cell membrane, cell wall, and cellular proteins; and peroxidation of lipids present within cell membranes [90]. Nitric oxide (NO) is another highly reactive free radical that quickly interacts with superoxide anion radicals to create another highly reactive molecule known as peroxynitrite (ONOO-), which consequently triggers harmful influences on the exposed organisms [95]. Normally, ROS can be diminished via modulation of the antioxidant enzyme system, which includes glutathione-S-transferase (GST), catalase (CAT), superoxide dismutase (SOD), glutathione reductase (GR), glutathione peroxidase (GSH-Px), and selenium GSH-Px (Se-GSH-Px), and non-enzymatic mechanisms such as reduced glutathione (GSH), and decreases the levels of malondialdehyde (MDA) to maintain redox homeostasis [87,96]. MDA is one of the breakdown products of lipid metabolism and is used as a dependable marker of lipid peroxidation (LPO); meanwhile; the primary biological function of SOD is the detoxification of the superoxide anions created by partial reduction of O_2_ and its transformation to hydrogen peroxide (H_2_O_2_) [97].

Herein, Sánchez-Muros, et al. [17] found a significant decline in hepatic SOD activities with no significant differences in hepatic CAT, GSH-Px, GR, and GST activities in Nile tilapia fed TM-based diets compared with those fed FM-based diets. In a similar sense, a significant reduction in hepatic SOD activity was also noticed in the Pearl gentian grouper fed diets with 18.75% of FM substituted with TM [65], and these authors also found the highest hepatic GR in groupers fed a diet with 6.25% of FM substituted with TM. Meanwhile, the lowest hepatic MDA levels were observed in groupers fed a diet with 18.75% of FM replaced with TM.

Su, et al. [20] demonstrated a significant elevation in serum SOD with a decline in plasma MDA levels in yellow catfish fed TM-based diets compared with the FM group. Differently, Xu, et al. [75] reported a significant elevation in hepatic MDA levels in mirror carps fed diets supplemented with a 25 g YMWO/kg diet for 59 days.

Henry, et al. [55] demonstrated considerably elevated SOD, CAT, GSH-Px, GR, and glucose-6-phosphate dehydrogenase (G6DP) activities with reduced MDA levels in the proximal and distal intestine of rainbow trout fed TM-supplemented diets. The authors suggested that TM could augment the antioxidant defense in the proximal and distal parts of the intestine. Henry, et al. [55] reported a significant decrease in serum nitric oxide (NO) levels in European sea bass groups fed TM-based diets with or without added digestive enzymes. A significant increase in SOD activity was reported in giant freshwater prawns fed diets with increased TM larvae protein levels [30].

Sankian, et al. [73] reported a significant elevation in serum GSH-Px activity in mandarin fish fed diets with 30% full-fat TM larvae at the expense of FM, with no considerable changes in serum SOD activity. Conversely, Jeong, et al. [22] reported no significant differences in serum SOD and GSH-Px activities in rainbow trout fed graded levels of full-fat TM larvae compared with the FM group. Given what has been mentioned above, the antioxidant boosting activity of dietary TM could be linked to their chitinous content or other bioactive compounds [94,98]. In our opinion, differences in the antioxidant capacity in different fish species fed TM-based diets should direct the vision of researchers to pay more time and conduct several additional research studies for a better understanding of the reasons and mechanisms of action of dietary TM in potentiation of the fish antioxidative status.

## 8. Immune Responses

Throughout their life, fish are exposed to a wide range of infectious agents, which might lead to high mortalities and significant economic losses if not conveniently controlled [99,100]. Thus, finding immune-potentiating products that can be easily used as feed additives is of great importance [101,102]. Generally, it has been widely noted that the immune responses of aquatic animals could be significantly influenced by feed additives [103,104]. This section discusses the immune-stimulant effects of dietary TM in fish and shrimp practical diets.

### 8.1. Immune Responses of Fish

Henry, et al. [55] showed no significant differences in serum ceruloplasmin, lysozyme (LYZ), and serum antibacterial activities against Gram-negative bacteria in rainbow trout fed diets supplemented with graded levels of full-fat TM larvae with significantly increased serum trypsin inhibition and myeloperoxidase (MPO) activity in yellow mealworm (YMW) groups. Jeong, et al. [22] also recorded the highest MPO activity in rainbow trout fed a diet with 7% of FM substituted with full-fat TM larvae. Moreover, these authors found that the highest LYZ activity was observed in groups fed diets with 14% and 28% of FM replaced with full-fat TM larvae. Contrarily, Valipour, et al. [54] found no substantial alterations in immunoglobulin M (IgM) levels and alternative complement pathways (ACH50) in trout groups fed TM-based diets compared with those fed FM-based diets.

Henry, et al. [55] illuminated that the inclusion of 24.75% full-fat TM larvae in European sea bass diets induced a significant decrease in serum ceruloplasmin and MPO activities, with no effect on serum LYZ activity compared with the controls. These authors also demonstrated a significant decline in serum bacteriolytic activity against Gram-negative bacteria and serum trypsin inhibition in fish groups fed diets with TM larvae and exogenous proteases. Basto, et al. [60] reported a considerable increase in peroxidase (PO) activity, with no effects on plasma ACH50 and LYZ activity in seabass fed diets supplemented with graded levels of defatted TM larvae.

Sankian, et al. [73] exemplified a significant elevation in serum LYZ activity in mandarin fish fed diets supplemented with 30% full-fat TM larvae at the expense of FM. Furthermore, the authors recorded no significant differences in serum MPO activity and total immunoglobulin after the feeding trial. Su, et al. [20] reported that *P. fulvidraco* fed diets with graded levels of TM showed significantly increased LYZ activity 24 h post-challenge with *Edwarseilla ictaluri* compared to the FM group. Interestingly, there was a significant elevation in serum IgM levels with the increase in TM levels. The authors also observed noticeable upregulation of *major histocompatibility complex 2*, *interleukin 1*, *IgM*, and *hepcidin* genes in TM-supplemented groups at the end of the experiment 24 h post-challenge. The highest relative percentage of survival (RPS) was noticed in the group fed diets supplemented with TM to replace 27% of FM in the diets. In another study, Ido, et al. [29] reported the highest RPS in red sea bream challenged with *E. tarda* when fed diets with 20% of FM replaced with defatted TM larvae for 56 days before the challenge.

### 8.2. Immune Responses of Shrimp

Generally, the shrimp innate immune responses are principally comprised of activation of the prophenoloxidase (proPO) system [105,106] and phenoloxidase (PO) activity [107], phagocytosis of foreign bodies by hemocytes, encapsulation, nodule formation, the release of antimicrobial peptides (AMPs) such as crustin [108], and cell agglutination [31], in addition to the critical role of β-1,3-glucan-binding proteins (BGBP) as constitutive plasma protein [109]. 

Choi, et al. [31] reported significant upregulation of *BGBP* and *crustin* genes in the hepatopancreas of Pacific white shrimp fed diets supplemented with dried super-TM to replace both 25% and 50% of FM. The authors also reported a significant upregulation of the *ProPO* gene in the hepatopancreas of white shrimp fed diets supplemented with TM to substitute 50% of FM in their diets with the highest total hemocyte count (THC) and relative percent survival (RPS) after being challenged with white spot syndrome virus. Moreover, Motte, et al. [71] illustrated that white shrimps fed diets supplemented with defatted TM larvae to replace 50% of FM showed significantly decreased shrimp mortality after being challenged with *Vibrio parahaemolyticus*. Interestingly, it was found that feeding defatted TM larvae showed a significant increase in the THC, hemolymph protein level (HPL), and PO activity before challenging with *V. parahaemolyticus*. Furthermore, Motte, et al. [71] further reported continued elevation of THC levels with a significant decrease in the HPL, and PO and improved clearance of hemolymph with increasing defatted TM larvae levels in shrimp diets following the challenge with *V. parahaemolyticus*.

Feng, et al. [30] found that the inclusion of graded levels of TM larvae protein in diets of giant freshwater prawns resulted in a positive and significant dose-dependent increase in the THC, LYZ and PO activities, and numbers of semi-granular cells, granular cells, and hyaline cells with increasing levels of TM larvae protein. These authors further reported that the inclusion of TM larvae protein in shrimp diets induced a significant dose-dependent upregulation of the expression of *ProPO*, *lipopolysaccharide- and β-1,3-glucan-binding protein*, *peroxinectin*, and *α2-macroglobulin* genes in the hemocytes of treated shrimps compared with the controls. Attractively, there was a significant dose-dependent improvement of phagocytosis of *Lactococcus garvieae* and RPS against *Aeromonas hydrophila* in shrimp groups fed diets supplemented with graded levels of TM larvae proteins compared with the FM group [30]. It was found that dietary TM protein could encourage the activation of the proPO immune-related proteins present in aquatic animals [76].

From the previously reviewed literature, discrepancies among the immune responses of different fish species to the effect of dietary insect chitin may be accredited to the differences in the chitin content in each tested meal. In conclusion, it was importantly noted that the immunostimulatory effects of TM might be, in part, accredited to their chitin content [20,25]. Indeed, growing evidence has shown that the immune-potentiating effects of chitin are found in both cell-mediated and humoral immunity of fish [110,111] and can improve the resistance of fish and shrimp against infectious pathogens [112]. Moreover, it was reported that chitin has significant effects on the activation of innate and adaptive immune responses, such as activation of immune cells and activate cytokine and chemokine production [113]. Another hypothesis showed that the protein content of TM contains AMPs of various molecular weights, which significantly increases the fish resistance to bacterial pathogens such as *A. hydrophila*, *L. garvieae*, *E. tarda*, and *E. ictalui* [108,114]. To state that TM-based diets would improve the immune responses of fish and shrimp, extensive research studies should be done to identify the mechanisms by which TM enhance and augment the immune response and improve the resistance against pathogens.

## 9. Intestinal Health Status

### 9.1. Digestive Enzymes

The activities of digestive enzymes, especially protease, amylase, and lipase, play pivotal roles in the digestion process, feed use, and fish growth performance [117,118]. Sánchez-Muros, et al. [17] reported the highest protease activity in the stomach and intestines of Nile tilapia fed diets supplemented with TM larvae to substitute 25% of FM, followed by diets supplemented with TM larvae to replace 50% of dietary FM, and both groups showed appreciably higher digestive enzyme activities than those fed FM-based diets. Moreover, significantly increased intestinal protease and amylase activities were noticed in rainbow trout fed TM-based diets with 60% FM substitutions [50]. Differently, Hoffmann, et al. [42] found no considerable differences in the intestinal amylase, lipase, and trypsin activities in sea trout fed diets supplemented with 20% enzymatically hydrolyzed and non-processed full-fat TM larvae compared with those fed basal diets.

### 9.2. Diversity of Gut Bacterial Communities

The gut microbiome plays a pivotal role in improving the digestion of nutrients, which positively affects the overall fish health [119]. *Firmicutes*, *Actinobacteria*, *Alteromonadales*, and *Bacteroidetes* are gut microbiota that can improve nutrient digestibility and enhance the intestinal immunity of fish via counteracting the effects of pathogenic bacteria by competitive inhibition mechanisms [120,121].

Notably, it is well known that dietary inclusion of protein sources such as soybean meal can modulate and reshape the fish gut microbiota [122]. The impacts of dietary inclusion of TM on the diversity and reshaping of gut bacterial communities have been evaluated in several fish species, for instance, rainbow trout [57,58], Siberian sturgeon [66], and European sea bass, and gilthead sea bream [57]. Józefiak, et al. [66] recorded the highest count of Enterobacteriaceae, *Clostridium leptum* subgroup *Cl. coccoides*–*Eubacterium rectale* cluster, and *Lactobacillus* sp./*Enterococcus* sp. in rainbow trout fed diets in which 20% of FM was replaced with full-fat TM larvae for 71 days. Moreover, Józefiak, et al. [66] reported a significant increase in *Bacillus* spp., *Cl. coccoides*–*Eubacterium rectale* cluster, *Enterococcus* spp., and *Carnobacterium* spp. Counts, with no significant effects on the counts of *Aeromonas* spp., *Cl. leptum* subgroup, *Lactobacillus* spp., and Enterobacteriaceae in Siberian sturgeon fed diets in which 15% of FM was replaced with TM larvae for 60 days.

Antonopoulou, et al. [57] studied the potential effects of dietary inclusion of full-fat TM larvae on the diversity of gut bacterial communities in three fish species: gilthead sea bream, European sea bass, and rainbow trout. These authors reported about 598 operational taxonomic units (OTUs) belonged to *Proteobacteria*, *Bacteroidetes*, *Firmicutes*, and *Actinobacteria* after dietary inclusion of 60% full-fat TM larvae. The authors also further illustrated that 60%, 62.2%, and 30% of OTUs were absent in the gut microbiome before FM substitution with TM larvae in European seabass, gilthead seabream, and rainbow trout, respectively. Regarding the diversity indices of the gut bacterial communities in rainbow trout, there was a fivefold elevation of the Simpson dominance D index and a twofold decline of the Shannon H index following FM replacement with full-fat TM larvae.

From the aforementioned findings, we recognized that the increased abundance of *Cl. coccoides* in the fish gut positively affects the homeostasis of the gut microbiome because these bacteria play an important role in maintaining the overall functions of the fish gut [58,66]. *Cl. coccoides* could act as a normal commensal of the fish gut microbiota; they constitute a normal anatomical barrier to preventing the colonization of pathogenic bacterium and play significant roles in the gut immunology and, therefore, maintain normal fish health [123]. Besides, *Lactobacillus* sp. and *Bacillus* sp. help to stimulate the fish immune response and increased nutrient digestibility [124,125]. 

Importantly, it was hypothesized that high-fat and high-carbohydrate diets are regarded as the main factors that induce a modification of gut microbial communities such as *Enterobacteriaceae* and *Lactobacillus* sp. [126]. In a similar way, it can be assumed that feeding diets supplemented with TM with a higher fat content is significantly linked to increased bacterial group populations in the treated fish.

Generally, it was found that the increased richness and diversity of gut microbial communities are considered positive and desired features because they are usually associated with a healthy status of the host [117]. Contrarily, the reduced diversity of the gut microbiota is frequently correlated with the incidence of diseases or any pathological disorders. These nutritional disorders probably occur because of reduced competition with pathogens invaders, therefore favoring colonization in the GIT of fish [127]. To summarize, based on this analysis, we concluded that dietary supplementation with TM mealworms is important for modulating the GIT micro-ecosystem in fish. Moreover, the added value from the increased abundance of the beneficially important gut microbiota in fish fed TM-based diets is helpful in the improvement of the digestibility of nutrients, enhance fish health, and improve gut immunity to prevent colonization by disease-causing pathogens.

## 10. Quality Traits of Fish Fillets

It is commonly known that fish diets significantly affect the sensory quality criteria of the fillets and features such as texture, color, nutritional quality, and lipid content [128,129]. This section discusses the impacts of feeding fish TM-based diets at the expense of FM on the morphometric properties and organoleptic characteristics (physical traits and chemical composition) of fillets. Iaconisi, et al. [18] reported no significant differences in slaughter traits, carcass yield, water-holding capacity, hardness, cohesiveness, resilience, gumminess, and adhesiveness in blackspot seabream fed diets supplemented with full-fat TM larvae to replace up to 50% of FM compared with the control group. In a similar sense, Iaconisi, et al. [52] found no significant differences in the physical characteristics of fillets, including the water-holding capacity, cooking loss, and sheer force of rainbow trout fed diets supplemented with up to 50% of full-fat TM larvae compared with those fed control diets. Based on these results, we suggested that dietary supplementation with TM does not affect the physical characteristics of fillets of the treated fish [18]. These findings also highlight that dietary supplementation with TM might not affect the preferences of human consumers for fish fillets. However, we suggest further research to better evaluate the effects of dietary TM on other fish species. 

The pH value of fish fillets is a significant factor in evaluating the quality of fish fillets and is regarded as a guide for fish fillet freshness [130]. Herein, Iaconisi, et al. [18] reported lower pH values in fish fillets of blackspot seabream fed a diet with 50% of FM replaced with full-fat TM larvae; meanwhile, no differences were demonstrated in fish fed FM-based diets and diets with 25% of FM replaced with TM larvae. In rainbow trout, no significant changes were noticed in the pH value of fish fillets between fish fed diets supplemented with up to 25% full-fat TM larvae and those fed FM-based diets [52]. 

It is well-known that postmortem glycolysis favors lactic acid accumulation in muscles and subsequently decreases the pH value of muscles. Thus, a marked increase in the lactic acid content in fish fillets and a decrease in the pH value after death are closely linked to higher activities of anaerobic glycolysis before death, and this phenomenon is regarded as a good indicator of the early stress of the affected fish before death [131]. Based on the previously mentioned data, Iaconisi, et al. [18] supposed that 50% of dietary FM replacement with TM in the blackspot seabream induced great stress that led to a decrease in the pH value of fish fillets of the treated fish; therefore, these authors suggested not including TM in the diets of blackspot seabream over 25% of FM substitution. 

Color is one of the most critical indices of the sensory quality of fish fillets and is usually used to evaluate the economic value of the feed ingredients and can noticeably affect consumer acceptance of the fillets [132]. It was noticed that blackspot seabream fed TM-based meals showed no changes in the color values of the skin dorsal location compared to those in the control group, while inversely, TM-based diets considerably affected the color of the skin at the ventral location of fish flesh [18]. In other fish species, no significant effects were observed on the color of the fillets of rainbow trout fed TM-based meals; however, the skin color of the dorsal region had a higher redness index in the FM group than in the fish group fed a diet with 25% of FM replaced with full-fat TM larvae [52]. 

As regard the impacts of TM on color changes, it is known that insects are considered good sources of pigments, especially β-carotene that belongs to carotenoids with a red-colored pigment [34], and its amount in TM larvae is about <200 μg/kg [34]. As is known, fish skin and flesh are the primary storage locations of carotenoids [133], and because fish do not have the ability of de novo synthesis of these carotenoids, their presence in fish tissues is closely linked to dietary supplementation. The levels of carotenoid could explain the change in the redness index of the skin color when fish are fed insect meals at 50% of FM substitution with TM larvae [18]. Another reason is that the presence of riboflavin as a yellow-colored pigment (8.1 mg/kg in TM) [34] helps increase the yellowness of the fish flesh of fish fed TM-based diets [18].

## 11. Challenges in the Use of *T. molitor* in Aquafeed

In 2020, the different market prices of TM were 8.4 to 9.4 USD/kg in China, 10.8 to 14 USD/kg in the United States, 12.9 to 20 USD/kg in Europe, and 65 to 70 USD/kg in South Korea [134]. TM are one of the alternative protein sources in the aquafeed industry. The tendency toward their use is increasing, and people are currently showing great interest in insect-based feeds. One of the limitations of insect meal use is that the price is still relatively high to be economically convenient to substitute dietary FM. However, increasing its production will decrease its price to get suitable mass production at a reasonable price in the near future.

Consumer acceptance must also be prioritized and taken into consideration for meat products obtained from insect-fed animals and fish. Reports from few studies have shown the willingness of consumers to buy meat products from animals fed regular diets more than those fed insect-based diets. From these studies, it was observed that the acceptance rates for meat products (e.g., poultry, pigs, fish, and cattle) are above average [135]. So, in this sense, *T. molitor* looks like a promising feed ingredient for fish and shrimp to replace costly protein sources in feed for economical and better growth performance.

Safety also must be considered when insect and worm meals are used in fish feed, because they may carry chemical compounds such as toxins produced by their exocrine glands [136]. Moreover, *T. molitor* can contain a toxin consisting of benzoquinone compounds exuded by the defensive gland [137]. Benzoquinone acts as a toxic metabolite for both human beings and animals, having a carcinogenic effect, and it is also able to intervene in cellular respiration, resulting in kidney damage [138]. However, it is not confirmed how much benzoquinone endures in *T. molitor* larvae after processing, including heating, drying, cleaning, and grinding. Thus, there is an urgent need to observe the levels of benzoquinone that could be tolerated in model animals. Hence, establishing a processing method that can curb benzoquinone toxicity in *T. molitor* larvae products is much needed. Another challenge is that the insect genes may also have resistivity against antibiotics [54]. This implies that insects may be contaminated with pathogens or even mycotoxins from contaminated food. Furthermore, from food and the nurturing habitat, insects can also be amassed with heavy metals in their tissues [139,140]. For example, high cadmium levels have been observed in animal kidney corns, along with food ingredients such as wheat, mussels, and rice [141]. In this sense, Mlček, et al. [142] reported that the accumulation of heavy metals from diet and the quantity of lead in *T. molitor* larvae were below the detection limit, but the amount of cadmium in the dry case of *T. molitor* larvae was 147 to 230 mg/kg, which is above the diet limits [142]. Accumulated contents of heavy metals in insects may be toxic for human beings and animals. Therefore, regular monitoring of the potential content of specific heavy metals might be needed before the use of TM larvae as a feed ingredient.

## 12. Concluding Remarks and Future Perspectives

The aquaculture industry depends mainly on producing nutritionally balanced aquafeed to meet the nutritional requirements of aquatic animals. In this sense, looking for non-traditional replacements for FM and FO is among the main challenges required to sustain aquaculture activities. This review describes the possible trials for inclusion of *T. molitor* in aquafeed and reports successful trials with attractive results. The dietary inclusion levels vary based on feeding behaviors and farming conditions. Most of the obtained results were quantitative traits, but further studies are urgently needed to clearly understand their real modes of action, digestion, and absorption in the digestive system of the fish using more descriptive and qualitative tools. Unfortunately, the literature shows no controlled efforts by applying advanced omics techniques associated with clarifying the exact effects of *T. molitor* on digestion, absorption, and metabolic functions in the entire body of aquatic organisms. The immunological responses, antioxidative capacity, and tolerance of aquatic species against stressors are always attributed to the influence of nutrients on the physiological pathways correlated with the feed quality. As a result, nutritionists can propose more strategies of including *T. molitor* in aquafeed for sustainability without lowering the performance and quality of aquatic organisms.

## Figures and Tables

**Table 1:** Main chemical constituents (% of dry matter (DM)) of the yellow mealworm (*Tenebrio molitor* (TM)) according to the previously published literature.

Main Chemical Constituents (% of DM)					Calcium and Phosphorus (g/kg)		TM Stages	References
Crude Protein	Crude Lipids	Crude Fiber	Ash	Moisture	Calcium (Ca)	Phosphorus (P)		
46.4	32.7	4.6	2.9	5.3	4.3	7.1	Larvae	Ravzanaadii, et al. [32]
44.72	42.48	-	3.69	2.43	0.168	3.19	Larvae	Siemianowska, et al. [35]
52.8	36.1	-	3.1	-	2.7	7.8	Larvae	Makkar, et al. [36]
58.4	30.1	3.5	8	-	-	-	Larvae	Barroso, et al. [11]
51.93	21.57	7.2	4.69	ND	-	-	Lavae	Bovera, et al. [37]
51.9	23.6	4.7	-	-	-	-	Full fat Larvae	Gasco, et al. [38]
53.0	3.6	3.1	26.8	ND	2.7	7.8	Dried larvae	Khan, et al. [14]
53.22	34.54	6.26	4.04	-	0.78	1.039	Larvae	Ghosh, et al. [39]
45.8	34.2	4.0	2.5	5.8	3.8	7.0	Dried Larvae	Hussain, et al. [40]
60.21	19.12	22.35	4.2	7.25	0.5	0.976	Larvae	Heidari-Parsa, et al. [41]
47.0	29.6	-	4.51	-	0.047	0.69	Dried larvae	Hoffmann, et al. [42]
51.3	26.0	8.21	7.14	-	-	-	Dried larvae	Gelinçek and Yamaner [43]

ND: Not detected.

**Table 2:** Essential and non-essential amino acid (AA) content of the yellow mealworm (*Tenebrio molitor*) (g/100 g of protein) according to the previously published literature.

Published Literature	Barroso, et al. [11]	Bovera, et al. [37]	Ghosh, et al. [39]	Khan, et al. [14]	Hussain, et al. [40]	Heidari-Parsa, et al. [41]	Hoffmann, et al. [42]
TM stages	Larvae	Larvae	Larvae	Dried larvae	Dried larvae	Larvae	Dried larvae
Amino acid content							
Essential AAs							
Isoleucine	5.87	2.63	1.98	4.6	4.51	1.83	1.99
Leucine	8.65	4.52	3.37	8.6	5.32	3.13	3.61
Lysine	6.03	1.68	2.01	5.4	4.51	2.50	2.53
Methionine	0.64	1.62	ND	1.5	1.34	0.52	0.70
Phenylalanine	4.29	ND	1.76	4.0	1.54	1.55	1.84
Threonine	4.49	2.71	1.83	4.0	1.64	1.70	1.87
Histidine	3.64	2.11	2.80	3.4	1.65	1.38	1.35
Valine	7.61	3.72	2.94	6.0	4.42	2.57	3.18
Tryptophan	-	1.75	-	0.6	-	-	-
Cystine	-	1.62	3.16	0.8	3.62	-	-
Tyrosine	4.18	-	3.45	7.4	2.32	-	3.00
**Non-essential AAs**							
Glycine	-	-	2.61	4.9	2.65	-	2.53
Proline	7.17	-	1.6	6.8	2.34	-	2.99
Arginine	6.14	3.61	2.23	4.8	2.21	2.23	2.40
Alanine	-	-	3.96	7.3	4.34	-	3.99
Serine	-	-	2.20	7.0	3.45	2.23	2.18

**Table 3:** Fatty acid composition of the yellow mealworm (*Tenebrio molitor*) (g/100 g of DM) according to the previously published literature.

Published Literature	Jeon, et al. [44]	Belforti, et al. [45]	Adámková, et al. [46]	Tzompa-Sosa, et al. [47]	Ghosh, et al. [39]
TM stages	Larvae	Full-fat larvae	Larvae	Larvae	Larvae
Saturated fatty acids (SFA)					
Lauric acid (C12:0)	0.21	-	0.30	0.23	0.11
Myristic acid (C14:0)	0.36	0.51	2.60	3.11	1.63
Palmitic acid (C16:0)	17.77	3.43	20.20	18.52	4.71
Stearic acid (C18:0)	3.23	0.64	4.30	2.43	0.08
Monosaturated fatty acids (MUFA)					
Oleic acid (C18: ln9)	39.71	-	37.76	49.7	15.56
Palmitoleic acid (C16:1n7)	1.41	0.40	0.40	2.79	0.89
Polyunsaturated fatty acids (PUFA)					
Linoleic acid (C18:2n6)	29.90	6.97	31.90	21.82	7.57
α-Linolenic acid (C18:3n3)	1.74	0.27	1.70	0.84	0.11
Eicosapentaenoic (C20:5n-3)	-	-	-	-	0
Docosahexaenoic (C22:6n-3)	-	-	-	-	-
Eicosenoic acid (C20: ln9)	-	0.31	-	-	0.02
Arachidonic acid (C20:4n6)	-	-	-	-	0.04
Docosatetraenoic acid (C22:4n6)	-	-	-	-	-
ΣSFA	21.76	4.94	28.30	24.29	6.94
ΣMUFA	41.357	8.01	38.10	52.51	16.58
ΣPUFA	31.63	7.24	33.60	22.66	7.78
Total fatty acids (TFA)	94.75	20.19	100	99.46	31.30
Σn3	1.739	0.27	1.70	0.84	0.11
Σn6	29.89	6.97	31.90	21.82	7.67
Σn3/Σn6 FA ratio	0.058	0.039	0.053	0.038	0.014

**Table 4:** Application and effects of using the yellow mealworm (*Tenebrio molitor*) in diets of salmonids/.

Salmonids		Dietary Yellow Mealworms			Effects	Recommended FM Replacement Level or Inclusion Doses	References
Fish Species	Initial Body Weight (g)	Worm Stage/Form	FM Replacement Levels OR Dietary Inclusion Doses	Duration (days)			
Rainbow trout (*Oncorhynchus mykiss*)	~115.9	Larvae	25% and 50% dietary inclusion	75	No effects on growth and body composition The lowest HSI values	Up to 50% dietary inclusion	Gasco, et al. [49]
	~5.01	Defatted larvae	20%, 30%, 60%, and 100% of FM replaced	60	↑ YMW levels = ↓ FCR, ↓ FI, ↑ FBW, and ↑ PER No effects on feed palatability	Up to 100% FM replacement	Saravanan, et al. [51]
	~115.6	Full-fat larvae	25% and 50% dietary inclusion (= 35% and 67% of FM replaced)	90	↑ Intestinal CAT, SOD, GPx, GR, and G6PD ↓ Intestinal MDA levels No effects on ceruloplasmin, LYZ activity, and NO levels Faster serum antibacterial activity ↑ Serum trypsin inhibition and MPO activity	Up to 50% dietary inclusion	Henry, et al. [55]
	~115.6 ~110	Full-fat larvae	Growth trial Digestibility trial	90 21	No effects on FBW, WG, and VSI ↓ HSI ↓ADC of CP in the 50% YMW group No effects on ADC of DM and CL ↑ CP and ↓ CL contents in fish fillets	Up to 50% dietary inclusion	Belforti, et al. [45]

			25% and 50% dietary inclusion				
	~115.6	Full-fat larvae	25% and 50% dietary inclusion	90	No effects on pH, water-holding capacity, cooking loss of fillets, fillet color, and proximate composition of fillets ↑ C16:0, C18:1n9, and C18:2n6 and ↓ EPA and DHA in fillets	Not higher than 25% dietary inclusion	Iaconisi, et al. [52]
	~115.6	Full-fat larvae	25% and 50% dietary inclusion	90	↑ Alanine, taurine, tyrosine, cysteine, leucine, and proline in fillets of fish in the 50% YMW group ↓ Glutamine, histidine, methionine, and threonine in fillets of fish in the 50% YMW group Lowest glycine and histidine in fillets of fish in the 50% YMW group	Not higher than 25% dietary inclusion	Iaconisi, et al. [53]
	~8.58	Live worms	15%, 30%, and 60% dietary inclusion of live worm	30	↑ Growth and PER with ↑ YMW levels ↓ FCR in the 60% YMW group Highest CP and CL in fillets ↑ Protease and amylase activities in the 60% YMW group	Up to 60% dietary inclusion of live worms	Harsij, et al. [56]
	~53.39	Full-fat larvae	20% dietary inclusion	71	↑ WG and No effects on FCR, SGR, PER ↓ Intestinal villus height Highest *Enterobacteriaceae*, *Cl. leptum* subgroup, *Cl. coccoides*–*Eubacterium rectale* cluster, and *Lactobacillus* sp.*/Enterococcus* sp.	20% dietary inclusion	Józefiak, et al. [66]
	~5.01	Defatted larvae	Dietary inclusion of 5%, 7.5%, 15%, or 25% (equal to 20%, 30%, 60%, and 100% of FM replaced)	90	↑ FBW, FI, SGR, and PER Better FCR No effects on body composition and ADC of DM, CP, CL, and GE ↑ Eetention of protein, phosphorus, and energy	Up to 100% FM replacement	Rema, et al. [48]
	~115.2	Full-fat larvae	60% dietary inclusion	90	598 OTUs belonging to *Proteobacteria*, *Bacteroidetes*, *Firmicutes*, and *Actinobacteria* 33% of OTUs in the gut absent before FM replacement	60% dietary inclusion	Antonopoulou, et al. [57]
	~10	Larvae powder	25%, 50%, 75%, and 100% of FM replaced	56	No effects on HSI, VSI, and SR% No effects on RBCs count, MCV, and MCHC ↓ Hb, CHO, and HTC with ↑ YMW levels No effects on ALT, AST, TG, GLU, HDL, and LDL No effects on IgM, cortisol, and ACH50	25% FM replacement	Valipour, et al. [54]
	~78.3 ~94.6	Partially defatted larvae	Growth trial Digestibility trial 5%, 10%, and 20% dietary inclusion (equal to 25%, 50%, and 100% of FM replaced)	154	No effects on SGR, FCR, PER, FI, IFBW, CF, and VSI No effects on ALT, AST, GDH, G6PD, ME, and FAS ↑ ADC of CP in control and 25% YMW groups No effects on ADC of DM, EE, and GE	20% dietary inclusion (100% FM replacement)	Chemello, et al. [21]
	~1.11	Full-fat larvae	7%, 14%, 21%, and 28% dietary inclusion	56	No effects on CF, VSI, HSI, body composition, and SR% No effects on ALT, AST, ALP, ALB, TP, TCHO, SOD, GPx, and TBIL Highest MPO in the 7% YMW group Highest LYZ in 14% and 28% YMW groups	14% dietary inclusion	Jeong, et al. [22]
Black sea trout (*Salmo trutta labrax*)	~2010 (♀) ~1607 (♂)	Dried larvae	25% of the weekly feed amount Two or three days a week	50	No effects on the number of eggs Egg diameters smaller in control than YMW groups ↑ Sperm volume and concentration in YMW groups	25% of the weekly feed amount before breeding	Gelinçek and Yamaner [43]
Sea trout (*Salmo trutta m. trutta*)	~0.14	Enzymatically hydrolyzed Or non-processed full-fat larvae	20% inclusion of YMW in diets (200 g/kg diet)	60	Lowest mortality and highest RGR ↑ HSI and VSI No effects on intestinal amylase, lipase, and trypsin activities No effects on hepatic ALT and AST	20% dietary inclusion	Hoffmann, et al. [42]

**Abbreviations:** ACH50: alternative complement; ADC: apparent digestibility coefficient; ALB: albumin; ALP: alkaline phosphatase; ALT: alanine aminotransferase; AST: aspartate aminotransferase; CAT: catalase; CF: condition factor; CL: crude lipids; CP: crude protein; DHA: docosahexaenoic acid; DM: dry matter; EE: ether extract; EPA: eicosapentaenoic acid; FAS: fatty acid synthase; FBW: final body weight; FCR: feed conversion ratio; FI: feed intake; FM: fish meal; G6PD: glucose-6-phosphate dehydrogenase; GDH: glutamate dehydrogenase; GE: gross energy; GLU: glucose; GR: glutathione reductase; GPx: glutathione peroxidase; Hb: hemoglobin; HDL: high-density lipoprotein; HSI: hepatosomatic index; HTC: hematocrit; IBW: initial body weight; IFBW: individual final body weight; IWG: individual weight gainl LDL: low-density lipoprotein; LYZ: lysozyme activity; MCHC: mean corpuscular hemoglobin concentration; MCV: mean corpuscular volume; MDA: malondialdehyde; ME: malic enzyme; MPO: myeloperoxidase; NO: nitric oxide; OTUs: operational taxonomic units; PER: protein efficiency ratio; PUFA: polyunsaturated fatty acids; RBCs: Rrd blood cells; RGR: relative growth rate; SFA: saturated fatty acids; SGR: specific growth rate; SOD: superoxide dismutase; SR%: survival rate percentage; TBIL: total bilirubin; TCHO: total cholesterol; TG: triglycerides; TP: total protein; VSI: viscerosomatic index; WG: weight gain; YMW: yellow mealworm; ↑ = increase; ↓ = decrease.

**Table 5:** Application and effects of using the yellow mealworm (*Tenebrio molitor*) in the diets of European sea bass (*Dicentrarchus labrax*).

Fish Initial bwt (g)	Dietary Yellow Mealworm (*T. molitor*)			Effects	Recommended FM Replacement Level or Inclusion Doses	References
	Worm Stage/Form	FM Replacement Levels or Dietary Inclusion Doses	Duration (Days)			
~5.23	Larvae	25% and 50% dietary inclusion	60	25% YMW group = no adverse effects on WG 50% YMW group = growth reduction with less favorable effects on SGR and FI No differences in PER, FI, and whole-body composition	25% dietary inclusion	Gasco, et al. [59]
~5.22	Full-fat larvae	25% and 50% dietary inclusion	70	Negative effects on FBW, WG, SGR, and FI in the 50% YMW group No effects on CP and EE of body Higher ADCs of CP in the 25% YMW group	25% dietary inclusion	Gasco, et al. [38]
~65.3	Full-fat larvae	24.75% dietary inclusion (replacing 36% of FM) with or without exogenous proteases or carbohydrases	42	↓ Serum ceruloplasmin, MPO, and NO levels in YMW with or without enzymes ↓ Serum bacteriolytic activity against Gram-negative bacteria in YMW with proteases No effects on serum LYZ ↓ Serum trypsin-inhibition in YMW groups with exogenous proteases	Inclusion of 24.75% YMW in diets	Henry, et al. [25]
~5.2	Full-fat larvae	50% of FM replaced	70	598 OTUs belonging to *Proteobacteria, Bacteroidetes, Firmicutes*, and *Actinobacteria* 60% of OTUs absent in gut before FM replacement	50% FM replacement	Antonopoulou, et al. [57]
~55	Defatted larvae	40%, 80%, and 100% of FM replaced	70	No effect on plasma ACH50 and LYZ ↑ Plasma PO activity	Up to 80% FM replacement	Basto, et al. [60]
~33	Defatted or full-fat larvae	20% dietary inclusion	12	Compared to BSF groups, YMW groups characterized by the highest ADC of DM, CP, total EAAs, CL, GE, and phosphorus	20% dietary inclusion	Basto, et al. [24]
~5.7	Whole larvae	30% of FM replaced	84	No effects on SR%, VSI, HSI, SGR, FI, and RGL No effects on serum ALT, AST, and GDH ↓ Serum GLU, CHO, and TG levels	30% FM replacement	Mastoraki, et al. [23]
~10.7	Larvae	50% of FM replaced	49	No effects on FCR and PER ↓ SGR, FI, DGC, and WG No effects on proximate composition of fillets	Lower than 50% FM replacement	Reyes, et al. [61]

**Abbreviations:** ACH50: alternative complement pathway; ADCs: apparent digestibility coefficients; ALT: alanine aminotransferase; AST: aspartate aminotransferase; BSF: black solider fly; CHO: cholesterol; CL: crude lipids; CP: crude protein; DGC: daily growth coefficient; DHA: docosahexaenoic acid; DM: dry matter; EAAs: essential amino acids; EE: ether extract; EPA: eicosapentaenoic acid; FBW: final body weight; FCR: feed conversion ratio; FI: feed intake; FM: fish meal; GDH: glutamate dehydrogenase; GE: gross energy; GLU: glucose; HSI: hepatosomatic index; LA: linoleic acid; LYZ: lysozyme activity; MPO: myeloperoxidase; NO: nitric oxide; PER: protein efficiency ratio; PO: peroxidase activity; PUFA: polyunsaturated fatty acids; RGL: relative gut length; SGR: specific growth rate; WG: weight gain; YMW: yellow mealworm; ↑ = increase; ↓ = decrease.

**Table 6:** Application and effects of using the yellow mealworm (*Tenebrio molitor*) in the diets of sparids (family *Sparidae*).

Sparids		Dietary Yellow Mealworm (*T. molitor*)			Effects	Recommended FM Replacement Level or Inclusion Doses	References
Fish Species	Initial bwt (g)	Worm Stage/Form	FM Replacement Levels or Dietary Inclusion Doses	Duration (days)			
Gilthead seabream (*Sparus aurata*)	~45	Larvae	25% and 50% of FM replaced	60	No adverse effects on WG and FBW with slight ↓ PER and FCR in the 25% YMW group ↓ Growth in the 50% YMW group No differences in whole-body composition	25% FM replacement	Piccolo, et al. [27]
	~105 (Exp. 1)	Full-fat larvae	25% and 50% dietary inclusion (equal to 35% and 71% of FM replaced)	163	Exp. 1 Higher FBW, SGR, WG%, and PER and lower FCR in the 25% YMW group Exp. 2 Lowest dressed yield % in the 50% YMW group Highest VSI values in the 50% YMW group	25% dietary inclusion	Piccolo, et al. [26]

	~87 (Exp. 2)						
	~105.2	Full-fat larvae	50% of FM replaced	163	598 OTUs belonging to Proteobacteria, *Bacteroidetes*, *Firmicutes*, and *Actinobacteria* 62.2% of OTUs in gut absent before FM replacement	50% FM replacement	Antonopoulou, et al. [57]
	~105.2	Full-fat larvae	25% and 50% dietary inclusion	163	Higher alanine, leucine, and lysine in the 50% YMW group ↓ Histidine, taurine, and phenylalanine in YMW groups Higher arginine, leucine, phenylalanine, glycine, histidine, and proline in the 50% YMW group	Up to 50% dietary inclusion	Iaconisi, et al. [53]
Blackspot seabream (*Pagellus bogaraveo*)	~171.25	Full-fat larvae	25% and 50% of FM replaced	131	No differences in FI, FCR, SGR, slaughter traits, and yield No effects on water-holding capacity, hardness, cohesiveness, resilience, and proximate composition of fillets ↓ pH in the 50% YMW group	25% FM replacement	Iaconisi, et al. [18]
Red seabream (*Pagrus major*)	22.8–27.3	Defatted larvae	25%, 40%, and 65% dietary inclusion (38%, 60%, and 100% of FM replaced) 5% and 10% dietary inclusion (10% and 20% of FM replaced)	28 56	Highest FBW, SGR, and WG in the 100% YMW group No effects on FCR and FI Higher RPS in the 10% YMW group after challenge with *Edwarseilla tarda*	Up to 100% FM replacement	Ido, et al. [29]

**Abbreviations:** FBW: final body weight; FCR: feed conversion ratio; FI: feed intake; FM: fish meal; FO: fish oil; OTUs: operational taxonomic units; PER: protein efficiency ratio; RPS: relative percentage survival; SGR: specific growth rate; VSI: viscerosomatic index; WG%: weight gain percentage; WG: weight gain; YMW: yellow mealworm; ↑ = increase; ↓ = decrease.

**Table 7:** Application and effects of using the yellow mealworm (*Tenebrio molitor*) in the diets of several other fish species.

Fish Species		Dietary Yellow Mealworm (*T. molitor*)			Effects	Recommended FM Replacement Level or Inclusion Doses	References
Fish Species	Initial bwt (g)	Worm Stage/Form	FM Replacement Levels or Dietary Inclusion Doses	Duration (Days)			
Rockfish (*Sebastes schlegeli*)	~150	Dried larvae	Mixing YMW with basal diet (30:70 ratio)	28	High digestibility for CP, CL, and GE	Mixing YMW with basal diet (30:70)	Jang, et al. [115]
	~2.6	Larvae	8%, 16%, 24%, and 32% dietary inclusion	56	↓ WG, and FBW in 32% YMW than control No effects on daily FI and PER No effects on TP, ALT, AST, TG, TBIL, ALB, and TCHO	Up to 24% dietary inclusion	Jeong, et al. [91]
	~3.11	Mealworm	8%, 16%, 24%, and 32% dietary inclusion with methionine or 32% dietary inclusion without methionine	56	↑ WG, PR, and SGR with ↑ YMW up to 16% ↓ WG, PR, and SGR with ↑ YMW over 16% to 32% Plasma TG level negatively affected by YMW No effects on serum TP, ALT, AST, ALP, and TCHO No effects on whole-body composition, fillet analysis, and EAAs	Not more than 16% dietary inclusion	Khosravi, et al. [72]
Zebrafish (*Danio rerio*)	Larval stage	Mealworm	20% and 30% of FM replaced	70	No impacts on LC-PUFAs such as ARA, EPA, and DHA	20% FM replacement	Tetlow [116]
Black sea bass (*Centropristis striata*)	~29.0	Mealworm	25%, 50%, 75%, and 100% FM replaced	121	↑ FBW, WG, FI, and SGR in the 25% YMW group Higher final length, length gain, and SGR length in the 25% YMW group than 75% and 100% YMW groups	25% FM replacement	Redman, et al. [67]
Mandarin fish (*Siniperca scherzeri*)	~20.8	Full-fat larvae	10%, 20%, and 30% dietary inclusion	56	No effects on FI, SR%, HSI, VSI, CF, and body composition No effects on TP, ALB, ALT, AST, ALP, and TG ↑ Serum LYZ, and GSH-Px in 30% YMW group No effects on SOD, MPO, and TIg	Up to 20% dietary inclusion	Sankian, et al. [73]
Mirror carp (*Cyprinus carpio* var. specularis)	~13.98	YMWO	25 g YMWO/kg	59	Compared to BSFLO-supplemented group No differences SR%, CF, GRL, HSI, KI, VSI, SI, and body composition ↑ Hepatic MDA levels No differences in ALB, TP, ALT, AST, ALP, TCHO, and TG ↑ Expression of hepatic *IL-1β*, and *TNF-α* genes	25 g/kg	Xu, et al. [75]
Pearl gentian grouper (*Epinephelus lanceolatus* ♂ × *E. fuscoguttatus* ♀)	~6.60	Mealworm	6.25%, 12.5%, 18.75%, 25%, and 31.25% of FM replaced	50	No effects on SGR andTGC ↓ Hepatic SOD and MDA in the 18.75% YMW group Highest RPS in the 18.75% YMW group	12.3% FM replacement in diet	Song, et al. [65]
Siberian sturgeon (*Acipenser baerii*)	~640	Larvae	15% dietary inclusion	60	No differences FBW, WG, FCR, SGR, PER, and SR% ↑ Thickness of muscular layer of intestine ↑ *Cl. coccoides*–*Eubacterium rectale* cluster, *Bacillus* spp., *Carnobacterium* spp., and *Enterococcus* spp.	15% FM replacement in diet	Józefiak, et al. [58]
Japanese flounder (*Paralichthys olivaceus*)	~287.5	Larvae	7%, 17%, and 27% of FM replaced	42	17% FM replacement group has ↑ WG and SGR and better FCR with no significant effect on HSI	17% FM replacement	Kim, et al. [64]
African catfish (*Clarias gariepinus*)	~5.1	Mealworm powder	20%, 40%, 60%, 80%, and 100% of FM replaced	49	Highest FBW, WG%, SGR, PER, and NPU% in the 20% YMW group Higher whole-body CP in the 20% YMW group 100% YMW = slight decline FBW, SGR, FER, PER, and NPU% No mortality among all groups Higher whole-body CL content No effects on carcass CP content	Up to 80% of FM replacement	Ng, et al. [62]
Yellow catfish (*Pelteobagrus fulvidraco*)	~10.04	Mealworm	9, 18 and 27 g/100 g diet (equal to 25%, 50%, and 75% of FM replaced)	35	↓ Plasma MDA levels ↑ Plasma SOD with ↑ YMW levels at end of experiment and 24 h post-challenge with *E. ictaluri* ↑ LYZ in YMW groups 24 h post-challenge with *E. ictaluri* ↑ Plasma IgM levels Upregulation of *MHC II, IL-1, IgM*, and *HE* in YMW groups at end of experiment and 24 h post-challenge ↑ RPS in the 27% YMW group post-challenge	75% FM replacement (18 g/100 g diet)	Su, et al. [20]
Common catfish (*Ameiurus melas* Raf.)	~0.248	Mealworm	50% of FM replaced	90	↓ FBW and SR% in YMW than FM group No effects on FCR	50% FM replacement	Roncarati, et al. [63]
Nile tilapia (*Oreochromis niloticus*)	~6.5	Larvae	25%, and 50% FM replaced	42	↓ FBW, DGC, FCE, PER, and PERc No effects on FI, CF, and whole-body composition ↓ Hepatic SOD activity No effects on hepatic CAT, GSH-Px, GR, and GST Highest protease activity in stomach and intestine in the 25% YMW group followed by the 50% YMW group	Up to 50% FM replacement	Sánchez-Muros, et al. [17]
Nile tilapia in biofloc	~2.08	Mealworm	5, 10, 15 and 20% dietary inclusion	42	↑ FWB, WG, SGR, FCR, his, and carcass composition (DM and EE) with ↑YMW levels	Up to 10% dietary inclusion	Tubin, et al. [74]

**Abbreviations:** ALB: albumin; ALP: alkaline phosphatase; ALT: alanine aminotransferase; ARA: arachidonic acid; AST: aspartate aminotransferase; BSFO: black solider fly oil; CAT: catalase; CF: condition factor; CL: crude lipids; CP: crude protein; DGC: daily growth coefficient; DHA: docosahexaenoic acid; EAAs: essential amino acids; EPA: eicosapentaenoic acid; FBW: final body weight; FCE: Feed conversion efficiency; FCR: Feed conversion ratio; FER: Feed efficiency ratio; FI: Feed intake; FM: Fish meal; GR: Glutathione reductase; GRL: General relative intestine length; GSH-Px: glutathione peroxidase; GST: glutathione-S-transferase; HE: hepcidin; HSI: hepatosomatic index; IFI: intra-peritoneal fat index; IgM: immunoglobulin M; *IL-1*: interleukin-1; *IL-1β*: interleukin 1 beta; KI: Kidney index; LC-PUFAs: Long-chain poly-unsaturated fatty acids; LR: Lipid retention; LYZ: Lysozyme; MDA: Malondialdehyde; MHC II: Major histocompatibility complex 2; MPO: Myeloperoxidase; MUFAs: monounsaturated fatty acids; NPU%: net protein utilization percentage; PER: protein efficiency ratio; PERc: protein-efficiency-ratio-corrected crude protein; PR: protein retention; PUFAs: polyunsaturated fatty acids; RPS: relative percentage survival; SGR: specific growth rate; SI: spleen index; SOD: superoxide dismutase; SR%: survival rate percentage; TBIL: total bilirubin; TCHO: total cholesterol; TG: triglycerides; TGC: temperature growth coefficient; TIg: total immunoglobulin; *TNF-α*: tumor necrosis factor alpha; TP: total proteins; VSI: viscerosomatic index; WG%: weight gain percentage; WG: weight gain; YMW: yellow mealworm; YMWO: yellow mealworm oil; ↑ = increase; ↓ = decrease.

**Table 8:** Application and effects of using ythe ellow mealworm (*Tenebrio molitor*) in shrimp diets.

Shrimp Species		Dietary Yellow Mealworm			Effects	Recommended FM Replacement Level or Inclusion Doses	Reference
Species	Initial bwt (g)	Worm Stage	FM Replacement Levels or Dietary Inclusion Doses	Duration (days)			
Pacific white shrimp (*Litopenaeus vannamei*)	~2.43	Larvae	25%, 50%, and 100% FM replacement	56	50% and 100% of FM-replaced groups with significant ↑ WG and SGR values and better FCR	Up to 100% FM replacement	Chung, et al. [68]
	~10.1 ~4.42	Dehydrated larvae	15% dietary inclusion (digestibility trial) 25%, 50%, 75%, and 100% of FM replaced (growth trial)	7 42	↑ADC of DM, GE, CP, and EAAs WG, SGR, FI, FCR, SR%, and PR not affected No effects on body CP ↑ Body CL 13% to 1.88% in YMW groups	Up to 100% FM replacement	Panini, et al. [70]
	~9.23	Dehydrated larvae	25%, 50%, 75%, and 100% of FM replaced	42	No effects on moisture, CP, and ash in muscles FM replacement = linear ↑ in CL content No change in color and firmness among groups	Up to 100% FM replacement	Panini, et al. [69]
	~2.39	Dried superworms	25%, 50%, and 100% of FM replaced	56	Higher WG and SGR and better FCR ↑ Expression of *BGBP* and *crustin* in 25% and 50% YMW groups ↑ *ProPO* expression in the 50% YMW group Highest RPS after WSSV challenge in the 50% YMW group Highest THC in the 50% YMW group	Up to 50% FM replacement	Choi, et al. [31]
	1.5–1.6	Defatted larvae	25%, 50%, 75%, and 100% of FM replaced	56	↑ FBW, WG, ADG, and best FCR in the 50% YMW group No effects on FI, PER, and SR% ↓ Mortality after *Vibrio parahaemolyticus* challenge in the 50% YMW group	50% FM replacement	Motte, et al. [71]
Giant freshwater prawn (*Macrobrachium rosenbergii*)	~3.26	YMW larvae protein	4%, 8%, 12%, and 16% dietary inclusion of YMW larvae protein	70	↑ THC, semi-granular, granular, and hyaline cells ↑ SOD, PO, LYZ, and ALP ↑ Expression of *ProPO, LGBP, PE*, and *α2-macroglobulin* in hemocytes ↑ Phagocytosis of *Lactococcus garvieae* and RPS against *Aeromonas hydrophila*	12% YMW protein	Feng, et al. [30]

**Abbreviations:** AAs: amino acids; ADC: apparent digestibility coefficient; ADG: average daily gain; ALP: alkaline phosphatase; BGBP: beta-glucan-binding protein; CF: condition factor; CL: crude lipids; CP: crude protein; DGR: daily growth rate; DHA: docosahexaenoic fatty acids; EAAs: essential amino acids; EPA: eicosapentaenoic fatty acids; FBW: final body weight; FCR: feed conversion ratio; FI: feed intake; FM: fish meal; GE: gross energy; HPL: hemolymph protein level; LGBP: lipopolysaccharide- and β-1,3-glucan-binding protein; LYZ: lysozyme activity; PE: peroxinectin; PER: protein efficiency ratio; PO: phenoloxidase activity; PR: protein retention; ProPO: prophenoloxidase; RPS: relative percentage survival; SGR: specific growth rate; SOD: superoxide dismutase; SR%: survival rate percentage; THC: total hemocyte count; WG%: weight gain percentage; WG: weight gain; WSSV: white spot syndrome virus; YMW: yellow mealworm; ↑ = increase; ↓ = decrease.

## Data Availability

This review article did not report any data.
